# Contrasting Local and Macroscopic Effects of Collagen Hydroxylation

**DOI:** 10.3390/ijms22169068

**Published:** 2021-08-23

**Authors:** Sameer Varma, Joseph P. R. O. Orgel, Jay D. Schieber

**Affiliations:** 1 Department of Cell Biology, Microbiology and Molecular Biology, Department of Physics, University of South Florida, Tampa, FL 33620, USA; 2 Department of Biology, Department of Physics, Department of Biomedical Engineering, Illinois Institute of Technology, Chicago, IL 60616, USA; orgel@iit.edu; 3 Department of Chemical and Biological Engineering, Department of Physics, Illinois Institute of Technology, Chicago, IL 60616, USA; schieber@iit.edu

**Keywords:** molecular dynamics, polymers, fibril assembly, collagen, hydroxylation

## Abstract

Collagen is heavily hydroxylated. Experiments show that proline hydroxylation is important to triple helix (monomer) stability, fibril assembly, and interaction of fibrils with other molecules. Nevertheless, experiments also show that even without hydroxylation, type I collagen does assemble into its native D-banded fibrillar structure. This raises two questions. Firstly, even though hydroxylation removal marginally affects macroscopic structure, how does such an extensive chemical change, which is expected to substantially reduce hydrogen bonding capacity, affect local structure? Secondly, how does such a chemical perturbation, which is expected to substantially decrease electrostatic attraction between monomers, affect collagen’s mechanical properties? To address these issues, we conduct a benchmarked molecular dynamics study of rat type I fibrils in the presence and absence of hydroxylation. Our simulations reproduce the experimental observation that hydroxylation removal has a minimal effect on collagen’s D-band length. We also find that the gap-overlap ratio, monomer width and monomer length are minimally affected. Surprisingly, we find that de-hydroxylation also has a minor effect on the fibril’s Young’s modulus, and elastic stress build up is also accompanied by tightening of triple-helix windings. In terms of local structure, de-hydroxylation does result in a substantial drop (23%) in inter-monomer hydrogen bonding. However, at the same time, the local structures and inter-monomer hydrogen bonding networks of non-hydroxylated amino acids are also affected. It seems that it is this intrinsic plasticity in inter-monomer interactions that preclude fibrils from undergoing any large changes in macroscopic properties. Nevertheless, changes in local structure can be expected to directly impact collagen’s interaction with extra-cellular matrix proteins. In general, this study highlights a key challenge in tissue engineering and medicine related to mapping collagen chemistry to macroscopic properties but suggests a path forward to address it using molecular dynamics simulations.

## 1. Introduction

A wealth of information is now available on the structure, assembly, and regulatory mechanisms of collagen in modulating tissue mechanics, cell interactions, and signaling [1,2,3]. Advances in tissue engineering have also enabled many new applications of artificially reconstituted collagen, including cardiovascular and corneal disorder treatment, nerve regeneration, and reconstruction of skin, tendons, and muscles [4,5,6]. Yet, a critical challenge remains: due to the various length-scales involved in collagen assembly and function, mapping causal pathways between basic molecular chemistry and tissue properties continues to be complicated. Not surprisingly, while numerous congenital point mutations have also been identified in collagen that lead to connective tissue disorders [7,8], their underlying molecular modes of action remain largely unknown [9,10,11]. Similarly, in tissue engineering, it remains challenging to rationally design next-generation collagen scaffolds with improved mechanical strengths and capacities to interact with and influence cell signaling [4,5,6]. Addressing these issues from a fundamental standpoint requires a better understanding of how local chemical perturbations connect to their macroscopic responses. Here we examine this inter-scale relationship in the context of hydroxylation of Type I collagen.

**Figure 1:**
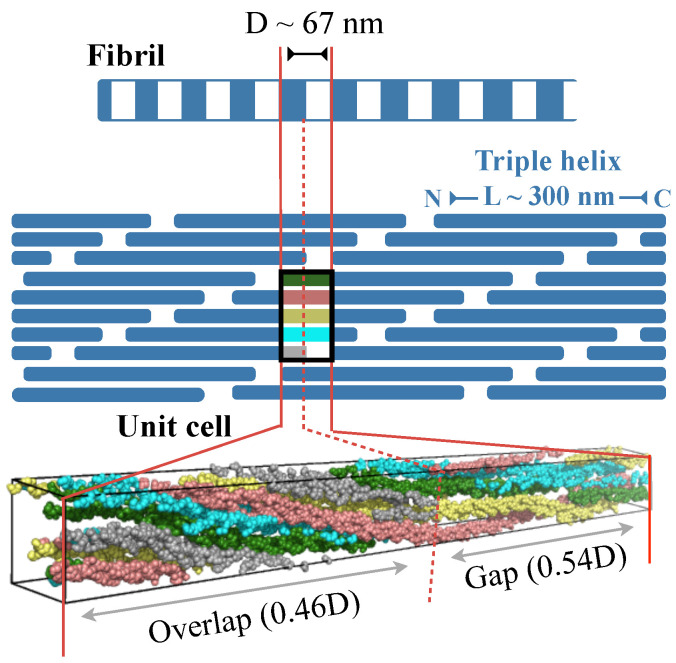
Structural hierarchy in Type I collagen fibril. The fibril is drawn at two different spatial resolutions, and a single unit cell or repeating unit of the fibril is also shown in molecular detail. The lower-resolution cartoon of the fibril depicts the repeating units (D-bands) containing low- and high-density regions, which emerge from the spatial arrangement of constituent triple helices (blue rectangles) in the fibril. The color scheme in the unit cell of the cartoon matches that in the unit cell showing molecular details.

Figure 1 illustrates the structural hierarchy in Type I collagen fibrils [1,2,3]. The building block, or monomer, of the fibril is a 300 nm long right-handed triple helix. In each of the three polypeptide chains in the monomer, every third residue is a glycine, which results in a repeating GXY sequence, where X and Y can be any amino acid. The monomers are arranged in a fibril is such a way that the N-termini of two closest monomers are axially staggered by D∼67 nm [12,13,14,15]. Additionally, the C-terminus of one monomer and the N-terminus of an axially adjacent monomer is separated by 0.54D. This staggered arrangement creates alternating regions of low and high protein density along the fibril axis with a repeating unit of length D. These D-periods or D-bands are, in fact, signature structural features of several collagen types. Fibrils, in turn, combine with other collagenous and non-collagenous molecules, such as proteoglycans, to form fibril bundles and fibers, which then assemble with each other to form tissue scaffolds.

Collagen is heavily hydroxylated [8,16,17,18,19]. X-ray diffraction studies on type-I collagen extracted from rat tails show that about 10% of its residues are hydroxylated [14,15]. Hydroxylation is found primarily on proline residues, and 297 out of the 376 prolines are hydroxylated. Additionally, 15 out of the 93 lysines were also found to be hydroxylated. Note that assignments of hydroxylated prolines and lysines in diffraction studies were made by using the amino acid sequence determined by Chapman and Hulmes [20], and considering that the differences in electron scattering factors for the hydroxylated residues are just under twice those of their corresponding non-hydroxlated forms [21].

Proline hydroxyls have been shown to be important to the stabilities of its constituent monomers [1]. Specifically, proline hydroxylation at the Y-position of the GXY sequence switches proline’s ring pucker preference from a Cγ-endo to a Cγ-exo conformation. This pre-organizes its main chain dihedrals to angles needed in the Y position for twisting into triple helices, effectively reducing the entropic cost associated with the twisting of peptides into triple helices [22]. Additionally, hydroxylation of prolines at either the X or Y position strengthens electrostatic interactions between individual polypeptides, which adds to triple helix thermal stability [23,24].

Proline and lysine hydroxylation also reduces triple helix hydrophobicity, which, from a fundamental standpoint, can affect fibril assembly. On the one hand, reduced hydrophobicity will make it energetically more difficult to extract a peptide from water and place it in a complex [25]. This effect will impede fibrillar assembly. On the other hand, increased density of hydrogen bonding groups could strengthen electrostatic interactions between triple helix monomers. In fact, analysis of our all-atom simulations of rat type I fibrils [26,27] indicates that the hydroxyl groups of hydroxylated prolines make up 21% of the hydrogen bonds between the triple helices in a fibril (inter-monomer hydrogen bonds). This latter effect will facilitate assembly and counter the former effect. Finally, reduced hydrophobicity can also increase sensitivity of assembly to solvent ionic strength [28].

Despite these demonstrated and presumed effects of hydroxylation, experiments show that even without hydroxylation, Type I collagen does assemble into its native D-banded fibrillar structure [29,30]. This raises two key questions. Firstly, how does such an extensive chemical change, which is expected to substantially reduce hydrogen bonding capacity, affect local structure? Secondly, how does such a chemical perturbation, which is expected to substantially decrease electrostatic attraction between monomers, affect collagen’s mechanical response?

To address these questions, here we carry out all atom molecular dynamics (MD) simulations of the rat type I collagen without hydroxylation and compare the results against our previous simulations of the hydroxylated state [26,27]. We have demonstrated previously [26,27] that our MD approach reproduces experimentally observed structural and mechanical properties of hydroxylated rat type I collagen. Additionally, MD simulations have also been employed to gain molecular insights into monomer stability [31], self-assembly of fibrils [31,32], inter-monomer cross-linking in fibrils [33], fibril elasticity [27], surface accessibility of protein binding sites [34], and relative orientations of monomers in fibrils [35].

## 2. Methods

### 2.1. Zero-Stress Simulations

We previously reported results from an MD simulation of the hydroxylated form of the rat fibril conducted under zero-stress boundary conditions [26]. The list of lysines and prolines that are hydroxylated are provided in the structure deposited in the protein data bank (PDB ID: 3HR2) [15]. This simulation is conducted on a single repeating unit of the fibril, but under periodic boundary conditions. Applying periodicity converts the unit cell into an infinitely wide and infinitely long fibril. Such a simulation setup lacks an interface between fibril and bulk solvent, and therefore, it represents the buried core of a fibril (Figure 1). The dimensions of the unit cell are exactly the same as that of the repeating unit observed in fiber diffraction [14,15]. It is a triclinic cell with the longest lattice constant equal to the D-band length of the fibril. This unit cell contains exactly one triple helix wrapped 4.46 times about the fibril axis, that is, the length of the triple helix L=4.46D. We note that simulating a single crystallographic unit cell under periodic conditions does not incorporate aperiodic variations across multiple unit cells. Nevertheless, because the unit cell we employ is the smallest periodic repeating unit observed in fiber diffraction studies [14,15], we consider it as an appropriate model of the fibril core.

The MD protocol we employed for simulating the rat fibril without hydroxylation is similar to what we employed for simulating the rat fibril with hydroxylation, except that here, instead of starting from the low-resolution fiber-diffraction structure [15], we start with a representative MD-equilibrated all-atom model of the hydroxylated fibril from our earlier work [26], which we set up using the fiber-diffraction structure. We dehydroxylated lysines and prolines in the MD structure and then subjected it to energy minimization. We then filled the remaining space with water (10,533 molecules), 100 mM NaCl, and 31 additional Cl${}^{-}$ ions to balance the net charge of the protein computed at physiological pH. We conducted another energy minimization and then subjected the system to a 20 ns MD simulation with unit cell dimensions kept fixed at their initial values. This step was carried out to allow waters to equilibrate, and we expected waters to equilibrate within this simulation time as it is more than two orders in magnitude longer than the residence times of waters at protein–water and protein–protein interfaces [36]. Next, we extended this trajectory by another 20 ns, but after releasing constraints on lattice dimensions and setting isobaric conditions where the trace of the Virial pressure tensor was set at 0.1 MPa. Finally, we extended this trajectory to 300 ns, but after setting a different isobaric boundary condition in which all the components of the Virial pressure tensor were regulated separately. The diagonal components were individually regulated at a pressure of 0.1 MPa, and the off-diagonal components were regulated at zero pressure to emulate a zero-shear condition. Equilibration in the final stage was monitored by following the time-evolution of lattice components, dihedral energy, and total potential energy, all of which level-off within 200 ns (Figure S1). We, therefore, use the final 100 ns for analysis.

### 2.2. Constant Strain Simulations

We also used all-atom MD to determine the fibril’s stress–strain relationship [27]. To accomplish this, we elongated the lattice constant parallel to the fibril axis (or the D-band length) by applying negative pressure, and then held it fixed at a length $D=D_{0}\left(1+\epsilon_{\left|\right|}\right)$, where $D_{0}$ is the D-band length under zero-strain ($\epsilon_{\left|\right|}=0$). No constraints were placed on other lattice components—the two remaining diagonal components were regulated at a pressure of 0.1 MPa and all off-diagonal components were regulated at zero pressure to emulate a zero-shear condition. We recorded the evolution of the axial stress, $\sigma_{\left|\right|}-\sigma_{0}$, where $\sigma_{0}=0.1$ MPa and $\sigma_{\left|\right|}$ is the Virial pressure along the fibril axis, as a function of simulation time, and the simulation was continued beyond $\sigma_{\left|\right|}$ equilibration. Each constant-strain MD simulation, therefore, yielded one point on the stress–strain curve. The simulation timescales for constant-strain simulations varied between 300 and 350 ns, and as can be expected, longer equilibration times were required for larger strains. The final 100 ns of the equilibrated constant-strain trajectories were then used for computing stress averages ($\langle \sigma_{\left|\right|}\rangle -\sigma_{0}$). Note that we have previously reported the stress–strain relationship for the hydroxylated form of the rat fibril [27], but the trajectory lengths here were longer, and here we extended them to match the trajectory lengths used for the stress–strain relationship of the un-hydroxylated form of the fibril.

### 2.3. MD Parameters

Water molecules are described using SPC/E parameters [37], and all other atoms are described using the Amber99sb-ILDN force field [38,39] that includes parameters for both hydroxylysine [26] and hydroxyproline [40]. In case of hydroxyproline, the force field parameters do exhibit preference for its ring’s Cγ-exo puckered conformation over its Cγ-endo conformation. Importantly, we have shown that the backbone dihedral energy landscape of this force field matches closely with our CCSD(T) reference map, and that this agreement is necessary to reproduce key structural properties of the fibril observed in experiments [26]. van der Waals interactions and direct space electrostatics are both truncated at 10 Å. Long-range electrostatics was computed using particle mesh Ewald [41] with a grid spacing of 1 Å. The bonds in proteins and the geometries of the water molecules are restrained, which permitted use of an integration time step of 2 fs. Temperature was regulated at 310 K using an extended ensemble approach [42] with a coupling constant of 0.1 ps. Pressure is regulated using a extended ensemble approach [43] with a compressibility of $4.5\times 10^{-5}$ bar${}^{-1}$ and a coupling constant of 1 ps. We used Gromacs v4.5 for all MD simulations [44].

## 3. Results and Discussion

To discern the effect of hydroxylation on equilibrium structural properties, we first compare results from zero-stress simulations of hydroxylated and un-hydroxylated fibrils. Next, to determine the effect of hydroxylation on mechanical properties, we compare results from constant-strain MD simulations.

### 3.1. Effect of Hydroxylation on Fibril Structure

Table 1 compares structural properties of fibrils simulated with and without hydroxylation. We note first that the D-band length of hydroxylated collagen is 66.21±0.03 nm, which is in close correspondence with estimates of ∼67 nm obtained from Atomic Force Microscopy, Transmission Electron Microscopy, and X-ray diffraction studies [1,2,3]. These simulations also reproduce the experimental observation [29] that hydroxylation has little effect on D-band length. Removal of hydroxylation slightly increases the D-band length to 66.54±0.02 nm.

Next we note that hydroxylation does not alter the gap fraction, which we determine from mass density variations along the fibril axis [27]. Hydroxylation also has a negligible effect on monomer widths and end-to-end distances. We define monomer width as the largest distance between the three nearest neighbor backbone nitrogen atoms of the three peptides. The widths listed in Table 1 are averages over the entire lengths of the triple helices, excluding the terminal telopeptide segments that are only partially twisted. The computed end-to-end distances, however, include the telopeptide segments.

Although hydroxylation has a minor effect on macroscopic structural properties, does it alter local structure? To address this, we first compute inter-monomer hydrogen bonds. There is no unique way to define a hydrogen bond [45], and for the rationale provided in our previous work where we analyzed protein–water and water–water hydrogen bonds [36], we used a geometric definition [46] in which the donor–acceptor distance is smaller than 0.35 nm, and the angle between the donor–acceptor and donor–hydrogen vectors is smaller than $30^{\circ }$. In the hydroxylated fibril, we find 351.4±1.4 inter-monomer hydrogen bonds in a single D-band unit (Table 1), and note that proline hydroxyls contribute to 21% of these hydrogen bonds. Hydroxylation removal results in a substantial drop (23%) in inter-monomer hydrogen bonding (Table 1). Expectedly, removal of hydroxylation also reduces protein–water hydrogen bonding. The average number of protein–water hydrogen bonds in a single D-band unit drops from 6346±38 to 5972±41. This amounts to a net 5.9% reduction in protein–water hydrogen bonding or 0.12 fewer hydrogen bonds per amino acid. In principle [25], reduced inter-monomer hydrogen bonding should negatively impact fibril assembly, but at the same time, reduced protein–water hydrogen bonding should facilitate fibril assembly.

**Figure 2:**
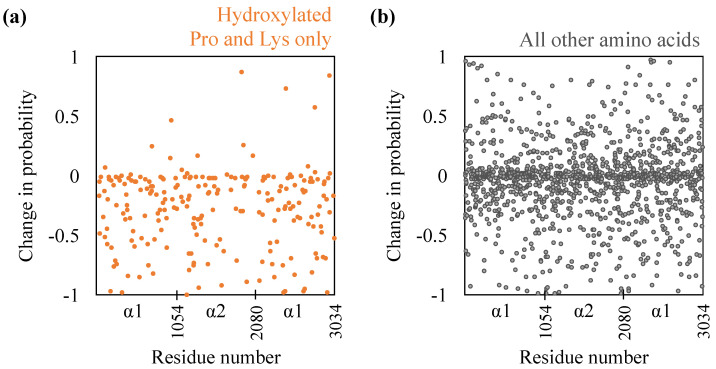
Effect of de-hydroxylation on inter-monomer hydrogen bonding probabilities (**a**) of only those prolines and lysines that are hydroxylated and (**b**) the rest of the amino acids. A negative value implies a decrease in probability due to hydroxylation removal. α1 and α2 refer to the two chain types in the triple helix.

While the extent of the drop in inter-monomer hydrogen bonding is comparable to that made by proline and lysine hydroxyls in the hydroxylated state, does it imply that the remaining inter-monomer hydrogen bonds remain unperturbed? To examine this, we compute the individual probabilities with which amino acids engage in inter-monomer hydrogen bonding. The results are shown in Figure 2. Firstly, as expected, for almost all hydroxylated prolines and lysines, hydroxylation removal reduces their hydrogen bonding probabilities. For some hydroxylated prolines and lysines, however, we do note that hydroxylation removal increases their hydrogen bonding probabilities. This implies that in these cases, other functional groups, including the backbone, are engaged in hydrogen bonding. Secondly, removal of hydroxylation, surprisingly, does substantially affect the hydrogen bonding probabilities of the remaining amino acids—probabilities decrease for 689 amino acids and increase for 504 amino acids. We also note from Figure 2 that the changes in probabilities are not restricted to any specific region of the D-band and are spread throughout the D-band. Nevertheless, we do note that the hydrogen bond probabilities of individual amino acids in the two hydroxylated states are moderately correlated, with a Pearson correlation of 0.47. This is not surprising because the helical paths of the monomers bring only certain regions into close enough proximity to make inter-molecular hydrogen bonds.

**Figure 3:**
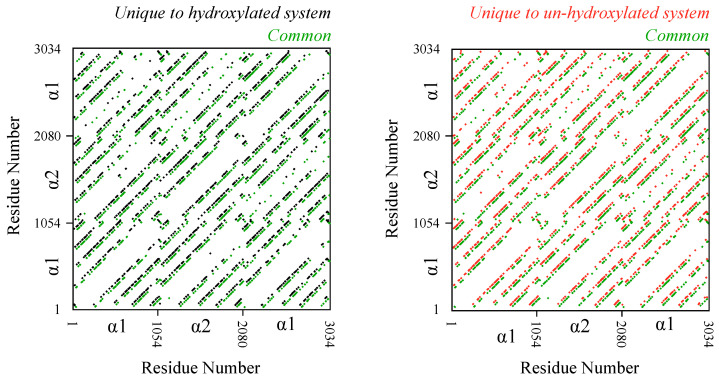
Effect of de-hydroxylation on residue pairwise contacts between monomers. Each inter-monomer contact is shown as a dot. Contacts common to the hydroxylated and un-hydroxylated systems are shown in green. Contacts unique to the hydroxylated system are shown in black, and those unique to the un-hydroxylated system are shown in red. Note that for the sake of clarity, the superimposed unique and common contacts are slightly shifted with respect to each other. Without the shift, diagonal patterns lie on top of each other. α1 and α2 refer to the two chain types in the triple helix.

The results above raise the question of the extent to which hydroxylation removal affects interfaces between monomers. To examine this, we compute and analyze inter-monomer residue contact pairs (Figure 3). We consider two residues to be in contact with each other if any of their heavy atoms are within 6 Å of each other for more than half the length of the trajectory. Trajectory analysis reveals that there are 3955 inter-monomer contact pairs in the hydroxylated system and 3831 inter-monomer contact pairs in the un-hydroxylated system. Comparison of the identities of these contact pairs reveals that 2229 pairs, or more than 50% of the pairs, are common to the two systems. This means that de-hydroxylation does alter local interfaces between monomers but not to the extent that the overall inter-monomer contact topology is altered. Additionally, just as we noted above in the case of hydrogen bonding, the changes to the contact interface are not restricted to any specific region in the D-band.

Taken together, we find contrasting effects of hydroxylation on fibril structure—while there are extensive local rearrangements at the molecular level, the macroscopic properties are only marginally affected.

### 3.2. Effect of Hydroxylation on Fibril Mechanics

To understand the effect of hydroxylation on mechanical properties, we determine stress–strain relationship along the fibril axis. Note that while no experimental estimates are available for the periodic fibrillar system we are simulating, we have previously compared our estimates for constituent triple-helix monomers against several different methodological approximations and experiments [27] and have shown that our employed MD parameters yield results within the experimental range [47,48,49].

**Figure 4:**
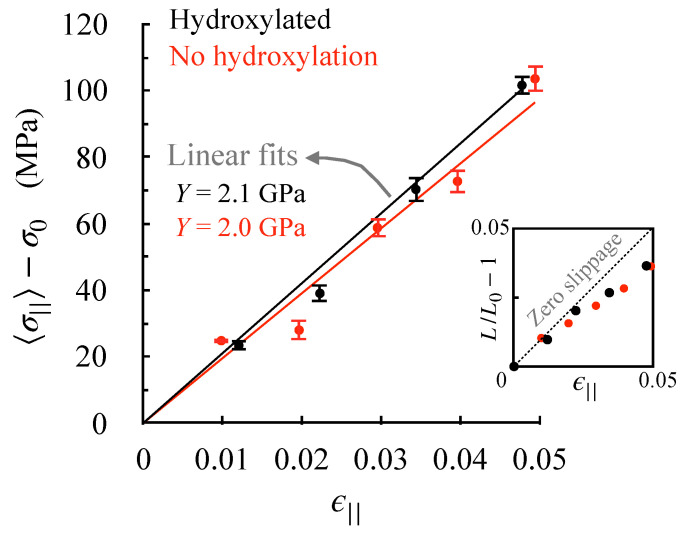
Effect of de-hydroxylation on axial stress–strain relationship. The data points are obtained from constant-strain ($\epsilon_{\left|\right|}$) MD simulations of the fibril. $\langle \sigma_{\left|\right|}\rangle -\sigma_{0}$ is the stress that builds up along the fibril axis, and the standard errors are obtained by block averaging. The inset shows the effect of strain on monomer end-to-end distance ($L/L_{0}-1$), where $L_{0}$ is the length of the monomer under zero-strain conditions. The standard deviations in $L/L_{0}$ are small (reported for $L_{0}$ in Table 1) and are, therefore, not shown.

As described in detail in the methods section, we obtain stress–strain relationships along the fibril axis by carrying out a set of separate MD simulations in which the lattice constant parallel to the fibril axis (or the D-band length) is held fixed at different strains ($\epsilon_{\left|\right|}>0$). We take the final 100 ns of each constant-strain trajectory to compute stress averages. The resulting stress–strain relationships for the two fibrils are shown in Figure 4. Assuming that strains smaller than 5% are in the linear (elastic) regime, we obtain Young’s moduli using Hooke’s law. We find that hydroxylation has little effect on the fibril’s Young’s modulus.

The inset in Figure 4 shows how the applied strain affects the monomer end-to-end distance—a linear dependence implies no slippage between monomers, and a negative deviation implies that monomers slide past each other under applied strain. In the assumed elastic regime, we note that straining induces some slippage between monomers, which increases with strain. This shows that the stress build-up in the elastic regime is associated primarily with an elongation of the triple helix. Elongation without slippage implies that windings in triple helices must be tightening, which is indeed the case—monomer widths at 5% strain reduce from 7.0 Å to 6.9 and 6.8 Å, respectively, in the hydroxylated and un-hydroxylated forms. Consequently, hydroxylation changes neither the Young’s moduli, nor the underlying basis for elastic stress build-up.

## 4. Conclusions

Collagen is the main protein in vertebrate tissue, and it is also among the most hydroxylated of proteins. To gain insight into the role of hydroxylation at the fibrillar level, and in general to understand how changes in collagen chemistry affect its macroscopic properties, here we carry out a comparative molecular dynamics study of hydroxylated and un-hydroxylated forms of rat Type-I collagen. Simulation of the hydroxylated form indicates that proline and lysine hydroxyls contribute to 21% of the hydrogen bonds between fibril monomers. Removal of these hydroxyls does result in an equivalent (23%) drop in hydrogen bonding. At the same time, de-hydroxylation also extensively rearranges the the hydrogen bond networks and local structures of non-hydroxylated amino acids. In contrast, there is only a minor effect of de-hydroxylation on macroscopic properties. Consistent with earlier experiments [29,30], de-hydroxylation minimally affects the D-band length. Other macroscopic structural properties, including gap-overlap ratio, monomer end-to-end distance and monomer width, are also minimally affected. The substantial drop in inter-monomer hydrogen bonding from de-hydroxylation could have affected the elastic response by reducing electrostatic attraction between monomers; however, the Young’s modulus also remains minimally affected. This finding suggests that inter-monomer hydrogen bonding plays only a minor role in elastic stress build-up, although it is plausible that there may be a lower limit to the extent of inter-monomer hydrogen bonding that is important to elastic response. Nevertheless, in both hydroxylated and un-hydroxylated fibrils, we do note that triple-helix windings tighten under stress and this increased tightening is what we expect contributes to stress build up in the elastic regime.

So why does de-hydroxylation affect macroscopic structural properties minimally, despite altering local structure extensively? Firstly, note that hydroxylation removal not only reduces inter-monomer hydrogen bonding, but also reduces protein–water hydrogen bonding. While the former effect would negatively impact fibril formation, the latter would reduce the monomer’s dehydration penalty and facilitate fibril formation (hydrophobic effect) [25]. It seems that increased hydrophobicity from de-hydroxylation does compensate for the drop in electrostatic attraction between monomers. Secondly, we note that de-hydroxylation extensively rearranges the inter-monomer hydrogen bonding network and local structure, and, therefore, it seems that this intrinsic plasticity in inter-monomer interactions precludes fibrils from undergoing any large changes in macroscopic properties. Collagen’s inter-monomer interactions have the capacity to substantially rearrange to maintain the fibril’s macroscopic structural integrity. We anticipate future studies to establish a more detailed causal link between macroscopic and local properties.

There are two key implications of our findings. Firstly, the observation that hydroxylation induces large changes in local structure suggests that hydroxylation can modulate interactions of collagen with other biomolecules in the cell and extracellular matrix [2,7]. Secondly, the observation that changes in chemistry can have marginal effects on macroscopic properties suggests that connective tissue disorders resulting from congenital mutations are not likely due to direct effects of mutations on fibril macroscopic properties; rather, the resulting mutational phenotypes in fibrillar and tissue structures are more likely due to changes in interactions of collagen with other biomolecules that facilitate assembly. This inference is supported by recent experiments [50,51] that show that while point mutations in collagen-like triple helices have little effect on triple helix structure, they do alter interactions of peptides with integrins and fibronectin. In the broader sense, these results provide a rationale for the inherent difficulty in deriving inter-scale relationships in collagen, but at the same time also exemplify how molecular simulations can be used to gain insight. 

## Figures and Tables

**Table 1:** Effect of hydroxylation on structural properties of fibrils. Standard deviations are obtained using block averaging.

Hydroxylation	Fibril D-Band Length (nm)	Fibril Gap-Fraction (%)	Monomer End-to-End Distance (nm)	Monomer Width (nm)	Inter-Monomer H-Bonds (1/V${}_{\mathrm{Unitcell}})$
Yes	66.21 ± 0.03	58.2 ± 0.1	290.0 ± 0.1	0.70 ± 0.001	351.4 ± 1.4
No	66.54 ± 0.02	58.9 ± 0.1	290.6 ± 0.1	0.70 ± 0.001	269.3 ± 0.9

## Data Availability

The data presented in this study are available on request from the corresponding author.

## Supplementary Materials

The following are available online at https://www.mdpi.com/article/10.3390/ijms22169068/s1.
