# Supplementary material for: Contrasting Local and Macroscopic Effects of Collagen Hydroxylation
Source: Int J Mol Sci. 2021 Aug 23;22(16):9068. doi: 10.3390/ijms22169068 (PMC8396666; doi:10.3390/ijms22169068)

Supporting Information:Contrasting local and macroscopic effects of collagen hydroxylation

Sameer Varma,[*,†] Joseph P.R.O. Orgel,[‡] and Jay D. Schieber[¶]

†*Department of Cell Biology, Microbiology and Molecular Biology, Department of Physics, University of South Florida, Tampa, FL 33620*

‡*Department of Biology, Department of Physics, Department of Biomedical Engineering, Illinois Institute of Technology, Chicago, IL-60616*

¶*Department of Chemical and Biological Engineering, Department of Physics, Illinois Institute of Technology, Chicago, IL-60616*

E-mail: svarma@usf.edu

Figure S1: Time evolution of unit cell dimensions ($|a|$, $|b|$, $|c|$, α, β and γ), total potential energy and dihedral energy of unhydroxylated system.

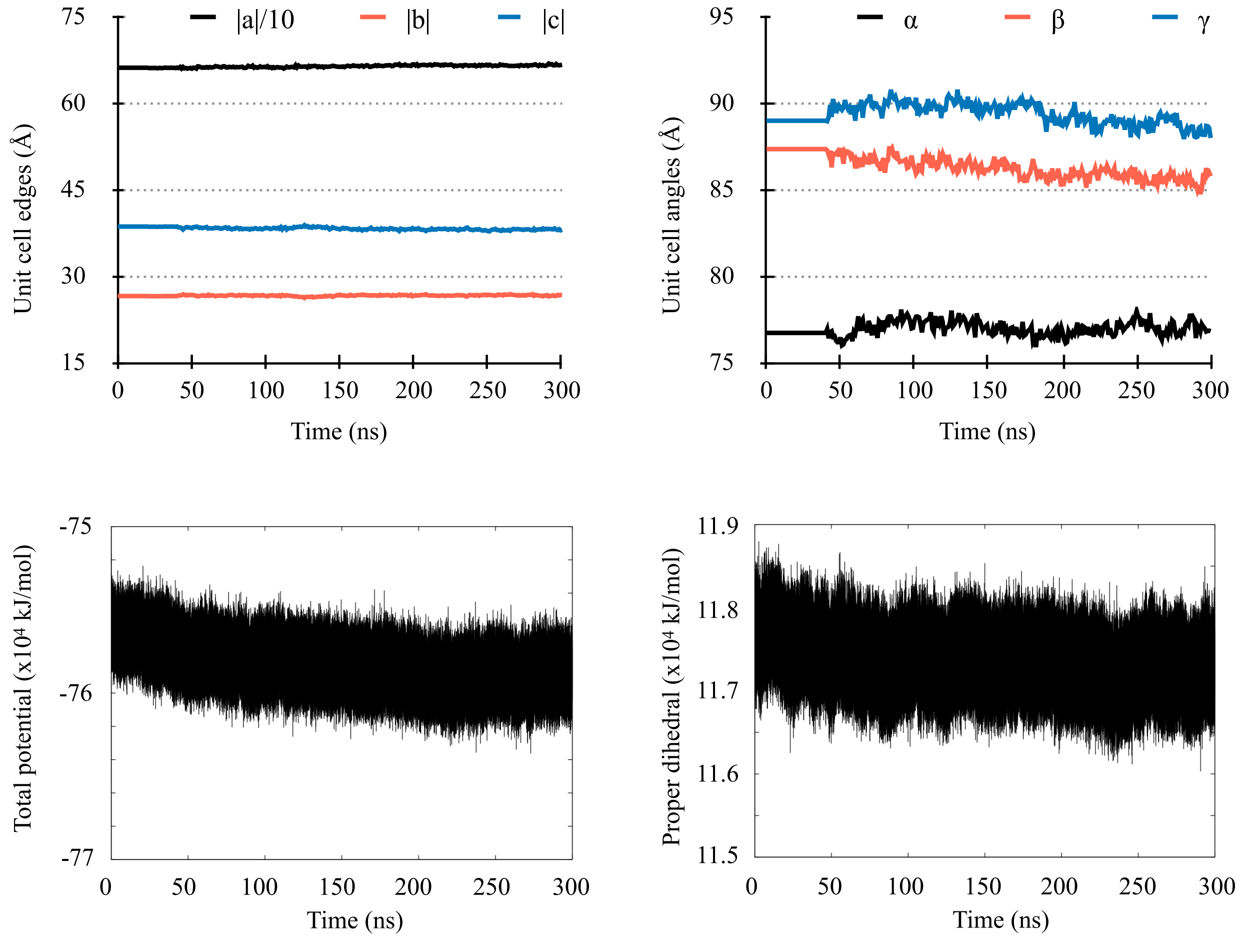

Supplement: Supplementary file 1 [file ijms-22-09068-s001.zip › ijms-1338612-supplementary.pdf]
